# Overexpression of N-terminal kinase like gene promotes tumorigenicity of hepatocellular carcinoma by regulating cell cycle progression and cell motility

**DOI:** 10.18632/oncotarget.2730

**Published:** 2015-01-10

**Authors:** Jian Wang, Ming Liu, Leilei Chen, Tim Hon Man Chan, Lingxi Jiang, Yun-Fei Yuan, Xin-Yuan Guan

**Affiliations:** ^1^ Departments of Clinical Oncology, The University of Hong Kong, Hong Kong, China; ^2^ State Key Laboratory for Liver Research, The University of Hong Kong, Hong Kong, China; ^3^ Center for Cancer Research, The University of Hong Kong, Hong Kong, China; ^4^ State Key Laboratory of Oncology in Southern China, Sun Yat-sen University Cancer Center, Guangzhou, China

**Keywords:** HCC, NTKL, CHD1L, dynamin2, metastasis

## Abstract

Amplification and overexpression of *CHD1L* is one of the most frequent genetic alterations in hepatocellular carcinoma (HCC). Here we found that one of CHD1L downstream targets, *NTKL*, was frequently upregulated in HCC, which was significantly correlated with vascular invasion (*P* = 0.012) and poor prognosis (*P* = 0.050) of HCC. ChIP assay demonstrated the binding of CHD1L to the promoter region of *NTKL*. QRT-PCR study showed that the expression of *NTKL* positively correlated with *CHD1L* expression in both clinical samples and cell lines. Functional study found that *NTKL* had strong oncogenic roles, including increased cell growth, colony formation in soft agar, and tumor formation in nude mice. Further study found that *NTKL* could promote G1/S transition by decreasing P53 and increasing CyclinD1 expressions. NTKL overexpression could accelerate the mitotic exit and chromosome segregation, which led to the cytokinesis failure and subsequently induced apoptosis. NTKL also regulated cell motility by facilitating philopodia and lamellipodia formation through regulating F-actin reorganization and the phosphorylation of small GTPase Rac1/cdc42. Using co-IP and mass spectrometry approach, we identified the large GTPase dynamin2 as an interacting protein of NTKL, which might be responsible for the phenotype alterations caused by NTKL overexpression, such as cytokinesis failure, increased cell motility and abnormal of cell division.

## INTRODUCTION

Hepatocellular carcinoma (HCC) ranks the fifth among the malignant cancers worldwide [1]. The molecular pathological mechanisms of HCC development have been widely studied during the last two decades. As reported by many studies, genomic aberrations have been frequently observed in HCC including gain of 1q, 6q, 8q, 17q and 20q, and loss of 4q, 8p, 13q, 16q and 17p have been frequently detected in HCC [2–5]. Among these genetic alterations, amplification of 1q21 is one of the most frequent changes in HCC and one candidate oncogene CHD1L has been identified [6]. The oncogenic function of CHD1L has been associated with its roles in inhibiting apoptosis [7], promoting tumor metastasis [8], and inducing resistance to chemotherapeutic drugs [9]. In *CHD1L*-transgenic mouse model, *CHD1L* could induce spontaneously tumors formation including HCC [10]. Recently, CHD1L has been shown to relocalize to DNA damage foci after DNA damage induction and regulate the DNA damage response [11].

As an SNF2-like family member, our previous study demonstrated that CHD1L could regulate expressions of its downstream target genes as a transcriptional factor [8]. Further study of the CHD1L-regulated genes would elucidate molecular pathogenesis of HCC. One CHD1L downstream target, N-terminal kinase like protein (*NTKL*, also called *Scyl1*), was investigated in the present study. *NTKL* is located at 11q13 with full-length protein containing 808 amino acids [12]. *NTKL* has been found to exhibit multiple subcellular localizations, including golgi apparatus, cytoplasm, centrosomes and nucleus [13]. Golgi NTKL has been reported to regulate golgi morphology and interact with Cop1 vesicles [14], while centrosome NTKL was reported to play a role in cell division [15]. In the present study, we found that NTKL, which was regulated by CHD1L, was frequently overexpressed in primary HCC cases. Functional assays showed that NTKL had strong oncogenic ability. Further study found that overexpression of *NTKL* might promote HCC tumorigenicity via regulating cell cycle progression and cell motility.

## RESULTS

### Expression of NTKL was regulated by CHD1L

**Figure 1:**
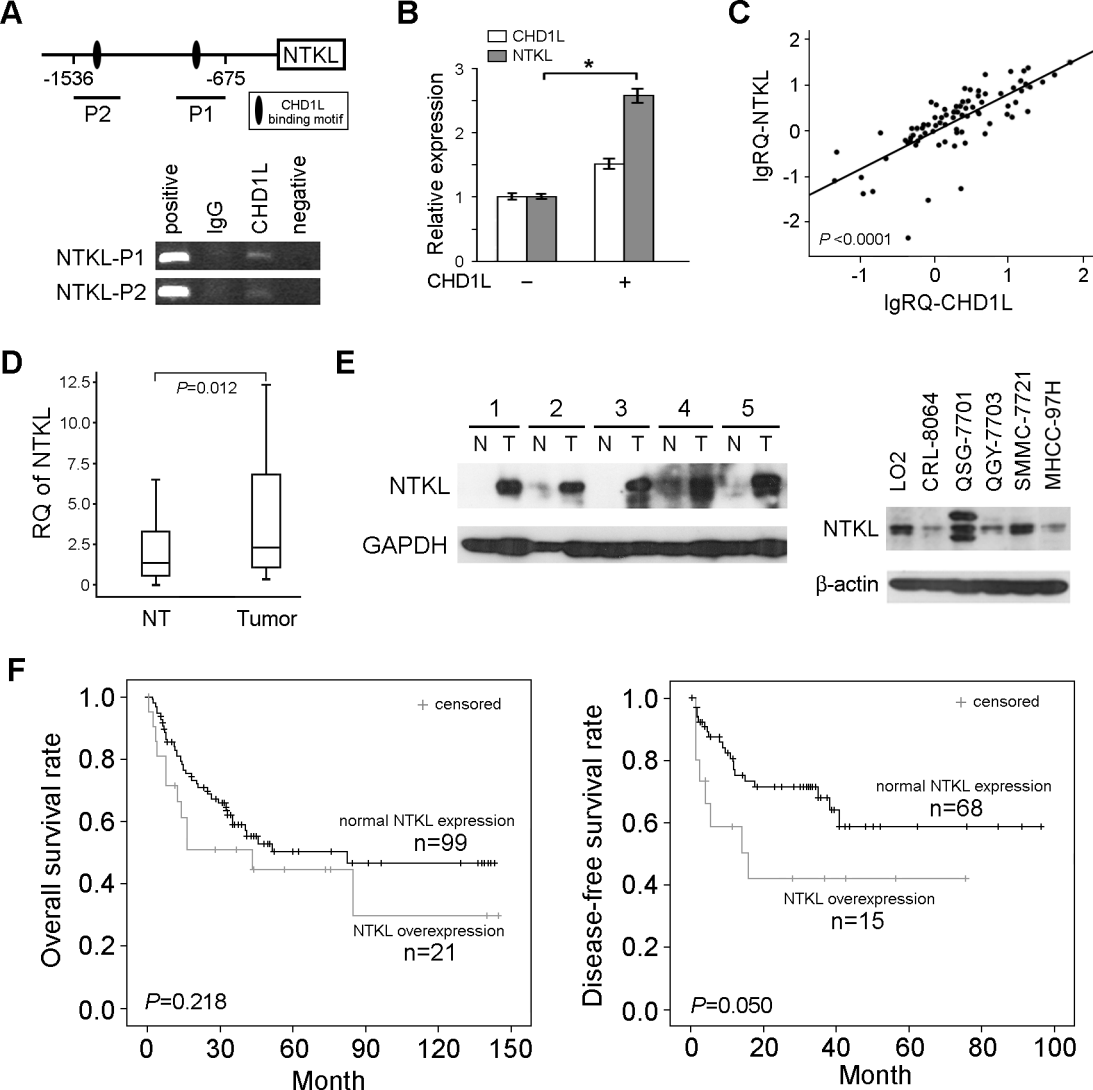
The CHD1L downstream target NTKL was frequently overexpressed in HCC **(A)** Two candidate CHD1L binding motifs were found at upstream (–1410~–1400 and –717~–707) of NTKL. The binding of CHD1L to the candidate motif sequences of NTKL was confirmed by ChIP assay. **(B)** QRT-PCR shows that overexpression of CHD1L in QGY-7703 cells could upregulate NTKL expression. **(C)** Expressions of *NTKL* and *CHD1L* were significantly correlated in 138 primary HCC samples detected by QRT-PCR. **(D)** NTKL expression was determined by QRT-PCR between tumor and corresponding non-tumor tissues in 138 HCCs, and the fold changes (RQ) was compared using T-test. **(E)** Expression of NTKL expression in primary HCCs (left) and HCC cell lines (right) was detected by Western blotting. **(F)** Kaplan-Meier survival analysis of the correlation between NTKL expression and HCC patient overall and disease free survivals.

Since our previous ChIP-based sequencing result suggests that CHD1L can bind to the promoter region of *NTKL* (data not shown), MatInspector was applied to predict potential CHD1L binding motifs within the promoter region of NTKL gene. Two candidate CHD1L binding motifs were found at upstream (–1410~–1400 and –717~–707) of NTKL (Figure 1A). ChIP assay was then used to confirm the binding of CHD1L to upstream DNA sequences of NTKL gene. The result showed that two DNA fragments NTKL-UP1 (–858~–675) and NTKL-UP2 (–1536~–1338) containing CHD1L-binding motifs were successfully detected in ChIP products precipitated by CHD1L antibody (Figure 1A).

To test whether CHD1L could upregulate NTKL expression, *CHD1L* was stably transfected into HCC cell line QGY-7703 cells. QRT-PCR result found that ectopic expression of CHD1L was able to increase NTKL expression (Figure 1B). To further confirm the correlation between NTKL and CHD1L, expressions of *NTKL* and *CHD1L* were evaluated in 138 primary HCC samples by QRT-PCR. A significant correlation between expressions of CHD1L and NTKL (*P* < 0.0001) was detected (Figure 1C).

### NTKL was frequently overexpressed in HCC

Since CHD1L is frequently overexpressed in HCC [6], the overexpression of NTKL in HCC was also examined by QRT-PCR in 138 HCCs. The result showed that the expression level of *NTKL* was significantly (*P* = 0.012) higher in HCC tumor tissues than that in non-tumor liver tissues (Figure 1D). Compared with paired non-tumor tissue, overexpression of *NTKL* (defined as 4-fold change) was detected in 24/138 (17.4%) of HCCs. Expression of NTKL at protein level was also checked in primary HCC cases and HCC cell lines by western blot assay (Figure 1E).

### NTKL overexpression was associated with invasion and poor prognosis in HCC

The clinical significance of *NTKL* overexpression in this HCC cohort was then evaluated by statistic analysis. The results found that *NTKL* overexpression was significantly associated with vascular invasion (*P* = 0.012), suggesting that *NTKL* might play roles in HCC metastasis (Table 1). Kaplan-Meier survival analysis showed that overexpression of *NTKL* was significantly correlated with poorer disease-free survival of HCCs (*P* = 0.050, Figure 1F). Cox regression analysis was carried out to further test the effects of *NTKL* on the prognosis of HCC patients. The result showed that overexpression of *NTKL* was significantly associated with poor 3-year survival rates of HCC patients both in univariable (*P* = 0.005) and multivariable analysis (*P* = 0.033, Supplementary Table 2).

**Table 1:** Clinico-pathological Correlation of NTKL Expression in 138 HCCs

Clinicopathological features	Number (n)	NTKL gene expression		P*
		Without overexpression	With overexpression	
**Gender**				
Male	116	96(82.7%)	20(17.3%)	
Female	22	18(81.8%)	4(18.2%)	1
**Age**				
< 60	111	93(83.8%)	18(16.2%)	
≥ 60	27	21(77.8%)	6(22.2%)	0.571
**HbsAg^†^**				
Negative	30	26(86.7%)	4(13.3%)	
Positive	90	72(80.0%)	18(20.0%)	0.674
**Serum AFP (ng/ml)**				
< 500	69	57(82.6%)	12(17.4%)	
≥ 500	50	40(80.0%)	10(20.0%)	0.812
**Tumor size(cm) ^‡, §^**				
≤ 5	36	32(88.9%)	4(11.1%)	
> 5	85	67(78.8%)	18(21.2%)	0.302
**Cirrhosis^§^**				
Absent	36	21(58.3%)	15(41.7%)	
Present	81	41(50.6%)	40(49.4%)	0.440
**Tumor encapsulation^§^**				
Absent	47	39(83.0%)	8(17.0%)	
Present	70	56(80.0%)	14(20.0%)	0.187
**Vascular Invasion**				
Negtive	71	59(83.1%)	12(16.9%)	
Micro	6	2(33.3%)	4(66.7%)	
Major	8	7(87.5%)	1(12.5%)	*0.012*
**Tumor stage(TNM)^§^**				
Stage I	54	46(85.2%)	8(14.8%)	
Stage II	30	24(80.0%)	6(20.0%)	
Stage III	34	27(79.4%)	7(20.6%)	0.738

* Two–sided χ^2^ test;

† Hepatitis B surface antigen;

‡ Tumor size was measured by the length of the largest tumor nodule;

§ Partial data is not available, and statistic was based on available data.

### NTKL had strong tumorigenic function

**Figure 2:**
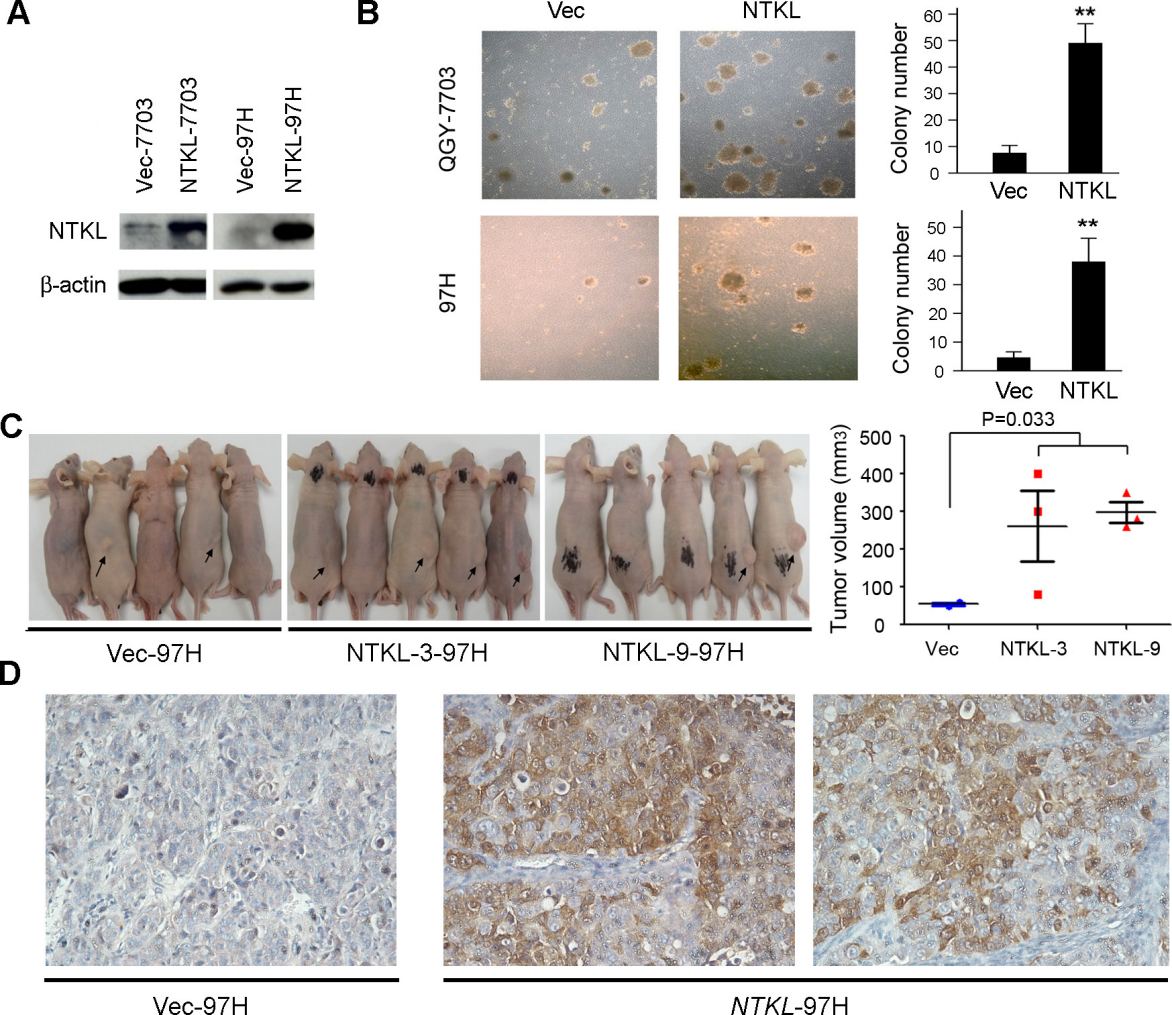
NTKL had strong oncogenic function **(A)** *NTKL* was stably transfected into HCC cell line QGY-7703 and 97H. Ectopic expression of NTKL was detected by Western blot. **(B)** Representative images of colony formation in soft agar induced by *NTKL*-transfected and Vec-transfected cells. The numbers of colonies were calculated and are depicted in the bar chart. The values indicate the mean ± SD of three independent experiments *P* < 0.001; independent Student's *t*-test). **(C)** Control cells and two clones of NTKL overexpressing cells (NTKL-3 and NTKL-9) were subcutaneously injected into nude mice. Representative examples of tumors (indicated by arrows) formed in nude mice injected with the indicated cells. The xenograft tumor volumes were shown in the right panel. **(D)** IHC staining of NTKL in the xenograft tumors.

To characterize the oncogenic ability of *NTKL*, full-length *NTKL* was cloned into expressing vector pCDNA3.1 and stably transfected into HCC cell lines QGY-7703 and MHCC-97H cells (*NTKL*-7703 and *NTKL*-97H). Empty vector-transfected cells (Vec-7703 and Vec-97H) were used as controls. Ectopic expression of NTKL in these transfected cells was confirmed by Western blot analysis (Figure 2A). *In vitro* soft agar assay showed that the number of colonies formed in soft agar were significantly increased in NTKL-transfected cells (*P* < 0.01, student's *t* test) compared with Vec-transfected cells in both QGY-7703 and MHC-97H cells (Figure 2B). To investigate the *in vivo* tumorigenicity of *NTKL*, tumor formation in nude mice was performed by injecting Vec-97H cells and NTKL-97H cells into 5 and 10 nude mice, respectively. After one month, tumor formations were detected in 2/5 and 6/10 mice induced by Vec-97H and *NTKL*-97H cells, respectively. Compared with xenograft tumors induced by Vec-97H cells, the tumor size was significantly larger in tumors induced by *NTKL*-97H cells (Figure 2C). Then xenograft tumors were subjected to IHC staining of NTKL and the results showed that NTKL overexpression could be detected in xenograft tumors induced by *NTKL*-97H cells (Figure 2D).

### Overexpression of NTKL promoted G1/S transition

**Figure 3:**
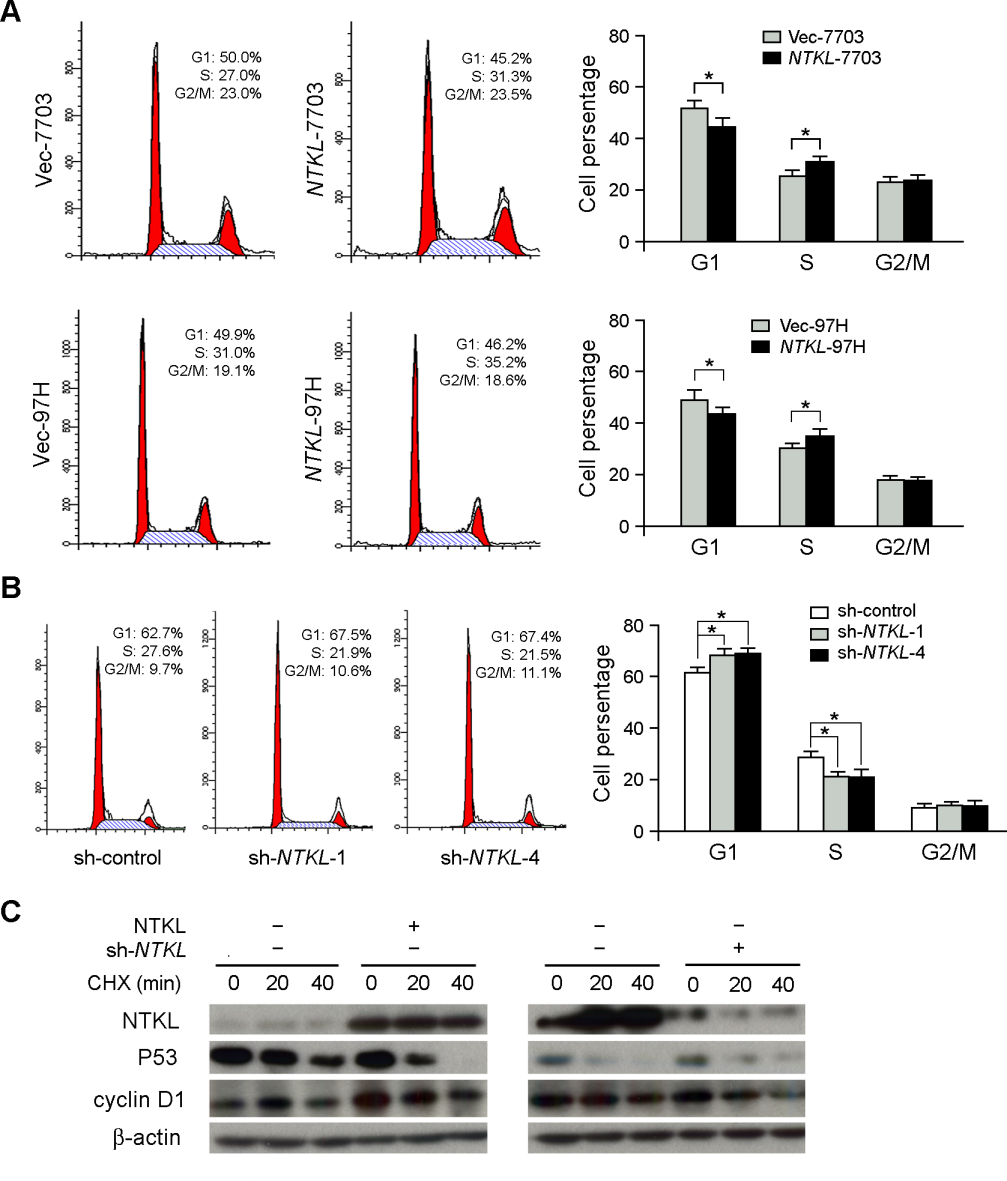
Overexpression of NTKL promoted G1/S transition **(A)** Flow cytometry was used to compare cell cycle distribution between *NTKL*-transfected and Vec-transfected cells. The percentages of cells at G0/G1, S and G2/M were summarized in bar charts. Data was shown as the mean ±SD of three independent experiments (*, *P* < 0.05; independent Student's *t*-test). **(B)** Depletion of NTKL by RNAi blocked cell cycle at G1 phase. Data were shown as the mean ±SD of three independent experiments (*, *P* < 0.05; independent Student's *t*-test). **(C)** The degradation of P53 and CyclinD1 was regulated by NTKL overexpression and depletion. Cells with NTKL overexpression or depletion were treated with CHX (50ug/mL) for time indicated to inhibit protein synthesis, and the degradation of P53 and CyclinD1 was observed by western blot.

To investigate the effect of *NTKL* on cell cycle, flow cytometry was used to compare proportions of cells in different cell cycle phases between *NTKL*- and vector-transfected cells. As shown in he results, cells in G1 phase decreased, while cells in S phase increased in *NTKL*-transfected cells, compared with control cells (*P* < 0.05, student's *t* test, Figure 3A). This indicated that overexpression of NTKL could promote G1/S cell cycle transition. To further confirm the results, two shRNAs (sh-*NTKL*-1 and sh-*NTKL*-4) specifically targeting NTKL were stably transfected into QGY-7703 cells. The non-targeting scramble shRNA (sh-control) was used as a control. Cell cycle distribution was analyzed by flow cytometry. The results showed that the percentages of cells in G1 and S phases were significantly increased and decreased, respectively, in *NTKL*-silencing cells (*P* < 0.05, student's t test), compared with cells treated with control shRNA (Figure 3B).

To further investigate the mechanisms of NTKL on cell cycle regulation, the effects of NTKL on cell cycle regulators P53 and cyclinD1 were examined by western blot. As shown in the results, overexpression of NTKL accelerated the degradation of P53, and enhanced the stability of cyclinD1. Conversely, knock down NTKL stabilized cyclinD1, and promoted the degradation of P53 (Figure 3C). This indicated that NTKL might regulate cell cycle progression through affecting protein stability of critical cell cycle regulators.

### NTKL overexpression accelerated the mitotic exit and chromosome segregation

**Figure 4:**
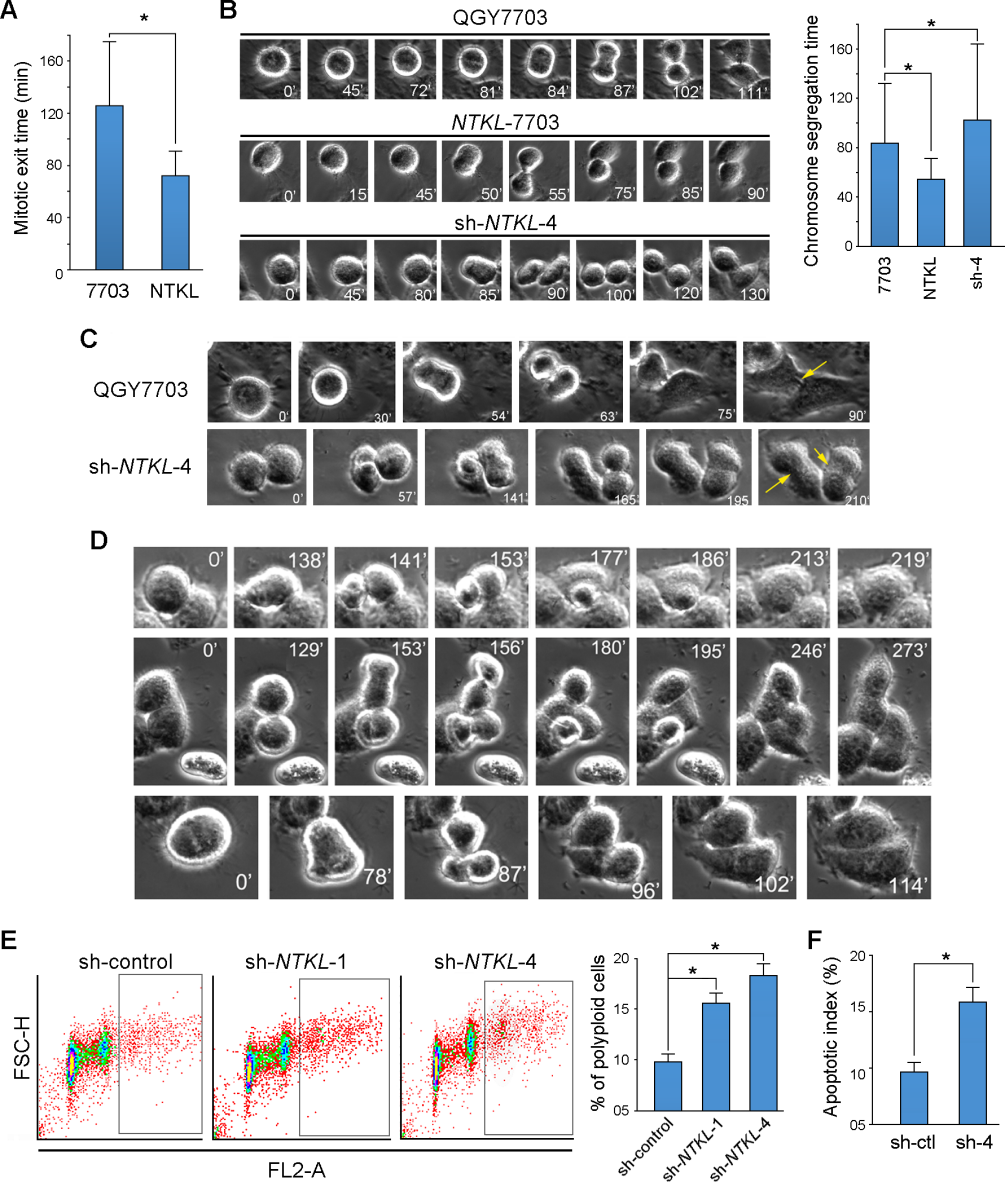
NTKL accelerated the mitotic exit and chromosome segregation **(A)** Time needed for mitotic exit was compared in QGY-7703 and NTKL-7703 cells, results were summarized in the bar-chart (*, *P* < 0.05; independent Student's *t*-test). **(B)** Time needed for chromosome segregation was compared among QGY-7703, NTKL-7703 and sh-NTKL-4 cells by real-time imaging analysis, and summarized in bar-charts (*, *P* < 0.05; independent Student's *t*-test). **(C)** Representative images of cell division with indicated cells captured by time-lapse microscopy. Midbodies were indicated by yellow arrows. **(D)** Representative images of cytokinesis failure in sh-NTKL-4 cells detected by time-lapse microscopy. **(E)** The percentage of polyploidy cells was detected by flow cytometry. Results were summairzed as the mean ±SD of three independent experiments (*, *P* < 0.05; independent Student's *t*-test). **(F)** The apoptotic index was compared between sh-control and sh-NTKL-4 cells by flow cytometry (*, *P* < 0.05, independent student's t test).

In addition to the G1/S acceleration, we also examined the effect of NTKL on cell mitosis by real-time imaging analysis. The time needed for chromosome segregation (from mitotic cell rounded up until the appearance of cleavage furrow) and the mitotic exit (from the rounded up of mitotic cells until daughter cells reattached) was compared among QGY7703, *NTKL*-7703 and sh-*NTKL*-4 cells. The results showed that the average time of mitotic exit needed for *NTKL*-7703 cells was much shorter than that of QGY-7703 cells (Figure 4A). In the sh-*NTKL*-4 cells, the mitosis was rarely completed, so that mitotic exit time of sh-*NTKL*-4 cells was not included in the comparison. For the chromosome segregation time, *NTKL*-7703 also showed the shortest time needed, while sh-*NTKL*-4 cells showed the longest time for chromosome segregation (Figure 4B).

### NTKL depletion induced cytokinesis failure and further cell apoptosis

When we look deeper into the cell cycle altered by *NTKL* depletion by real time imaging, QGY-7703 cells showed normal cell division. As indicated by yellow arrows, midbodies appeared normally after mitosis (Figure 4C). However, for the sh-*NTKL*-4 cells, midbody formation was diminished, and cells stuck together long after the mitosis. Daughter cells could not separate far from each other, and cell boundaries cannot be seen clearly (Figure 4C). Further observations found that cytokinesis failure was induced in sh-*NTKL*-4 cells. As shown in the first row of Figure 4D, sh-*NTKL*-4 cells entered mitosis and finished chromosome segregation 138 minutes later as indicated by the cleavage furrow formation. However, the following cytokinesis failed as indicated by the re-fusion of two daughter cells (Figure 4D, first row). Another kind of cytokinesis failure was shown in the second row of Figure 4D. Two incompletely divided, highly synchronized cells went on a second round mitosis before finishing cytokinesis. Due to the cytokinesis failure, four daughter cells stuck together sharing the interconnected cytoplasm with separated nucleus. In addition, multipolar cell division was also observed in sh-*NTKL*-4 cells by real time imaging. A mitotic cell went through mitosis in the presence of three polars, but resulted in only two daughter cells (the third row of Figure 4D). All the above phenotypes shown in Figure 4D were rarely observed in the QGY-7703 cells. The increased percentage of polyploidy cells induced by *NTKL* depletion was further confirmed by flow cytometry (Figure 4E). Cytokinesis failure usually will result in cell apoptosis^16, 17^. In the present study, enhanced cell apoptotic index was also observed in sh-*NTKL*-4 cells compared with sh-control cells (*P* < 0.01, student's *t* test) as detected by flow cytometry (Figure 4F).

### Overexpression of NTKL increased cell motility

**Figure 5:**
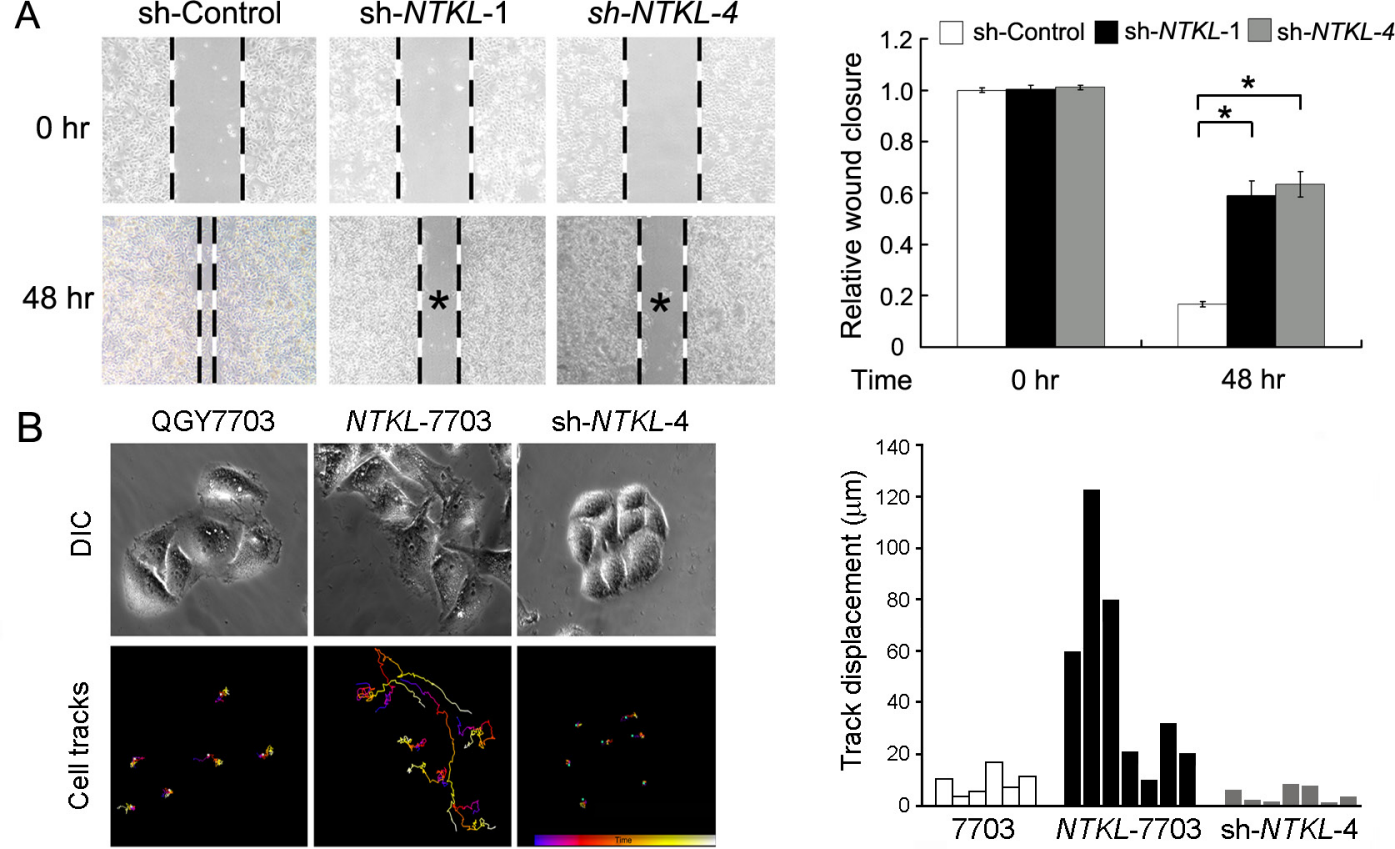
NTKL increased cell motility **(A)** Cell migration ability of sh-Control, sh-NTKL-1 and sh-NTKL-4 cells were detected by wound healing assay. *P < 0.05, independent student's t test. **(B)** Six to seven cells in one area of QGY-7703, NTKL-7703 and sh-NTKL-4 cells were observed for the migration analysis. Imaris software was applied for locating the center of each nucleus for the drawing of cell track in 300mins. Upper panel showed the cells in each group captured at the start point. Lower panel showed cell track that record the paths cells moved (blue ends, starting points; white ends, ending points). Track displacement of each cell was shown in bar chart.

Considering the multiple localization and function of NTKL, we then examined whether overexpression or depletion of *NTKL* would affect cell migration and spreading. As shown in Figure 5A, knock down of NTKL significantly decreased the migration ability of HCC cells in wound healing assay. Cell migration and motility observed from the real time imaging was also analyzed by Imaris software. *NTKL*-7703 cells showed the longest migration distance while sh-*NTKL*-4 cells almost did not move at all during the 300 mins recorded (Figure 5B). Cell movement paths were shown in the lower panel of Figure 5B. The blue ends represented the starting points and the white ends represented the ending points of cellular movement. We then quantitatively analyzed the cell migration by measuring the track displacements (the length of a straight line drawn from the starting to the ending point) with Imaris software. Results further proved the increased cell motility of *NTKL*-7703 cells as well as the reduced cell movements of sh-*NTKL*-4 cells (Figure 5B). These results indicated that NTKL can increase cell motility.

### NTKL enhanced cell motility by regulating F-actin reorganization

**Figure 6:**
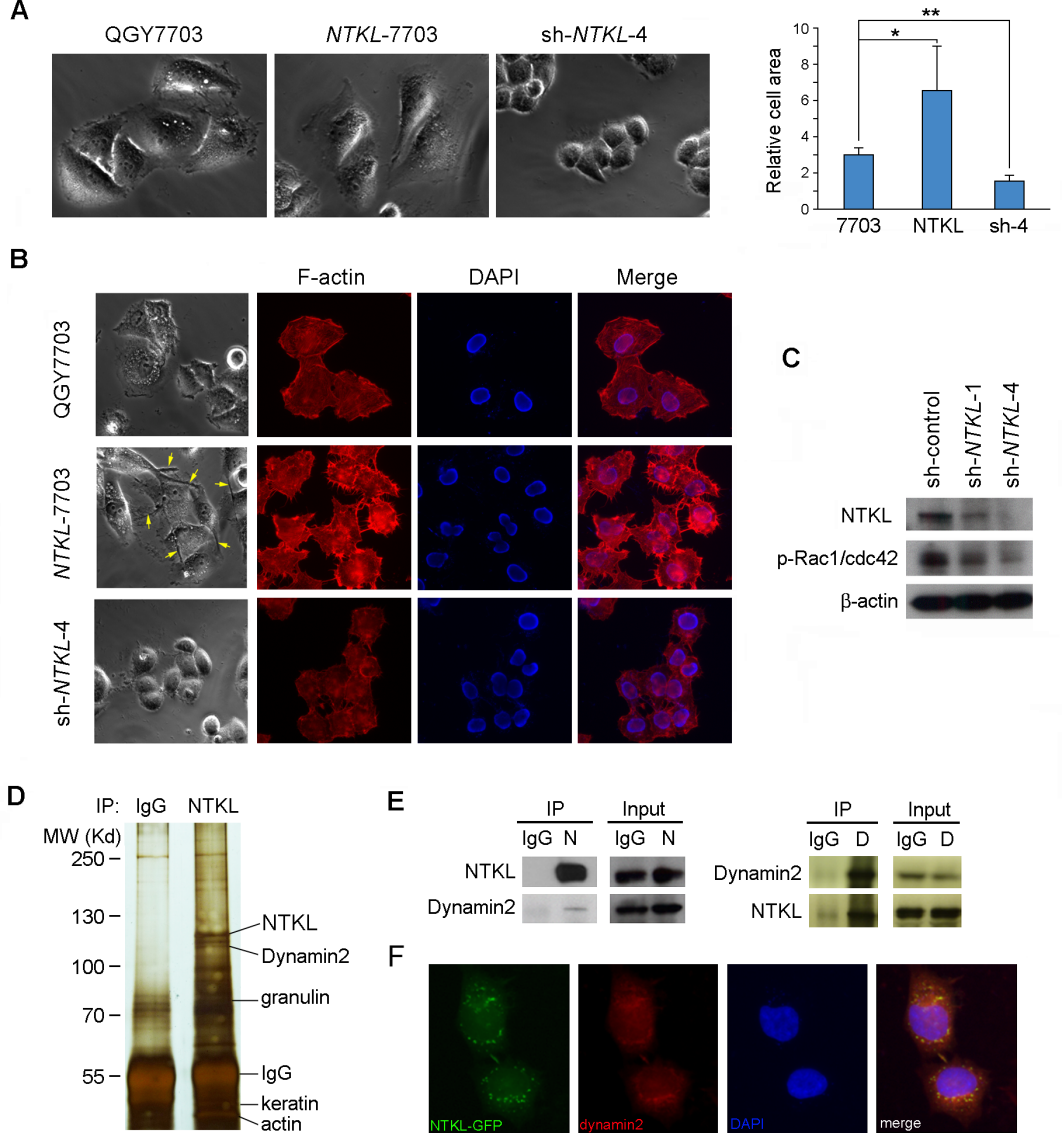
NTKL regulated the reorganization of actin cytoskeleton and interacted with dynamin2 **(A)** Representative images showing the morphology of QGY-7703, NTKL-7703 and sh-NTKL-4 cells. The cell areas were measured by image J and results were summarized in the bar chart (*, *P* < 0.05, **, *P* < 0.001). **(B)** Invasive structures (yellow arrows) composed of actin filaments were found in NTKL-7703 cells, but not in QGY-7703 and sh-NTKL-4 cells. Phalloidin staining indicated F-actin organization in QGY-7703, NTKL-7703 and sh-NTKL-4 cells. **(C)** Reduced p-Rac1/cdc42 was detected in NTKL depleted cells by Western blotting. **(D)** Results of Co-IP assay using NTKL and IgG (control) antibodies. Proteins recognized by mass spectrometry were indicated. **(E)** Interaction between NTKL and dynamin2 was confirmed by Co-IP assay using NTKL and dynamin2 antibodies. N, NTKL; D, dynamin2. **(F)** Colocalization of NTKL and dynamin2 was confirmed by immunofluorescence staining.

The morphological changes of *NTKL* overexpressing cells (*NTKL*-7703) and the *NTKL* depleted cells (sh-*NTKL*-4) were observed under microscope (Figure 6A). Compared to the QGY-7703 cells, *NTKL*-7703 cells showed a relatively more flattened morphology, while sh-*NTKL*-4 cells showed a rounded-up look. Cell areas of QGY-7703, NTKL-7703 and sh-*NTKL*-4 cells were quantitatively measured by image J. As indicated by the bar chart, the average cell area of NTKL-7703 was much larger than QGY-7703 cells, which was in accordance with the morphological observation. The sh-*NTKL*-4 cells showed the smallest cell area among the three cells (Figure 6A). Interestingly, elongated actin bundles were only observed in *NTKL*-7703 cells, but not in QGY-7703 and sh-*NTKL*-4 cells (Figure 6B), suggesting that *NTKL* could induce actin reorganization. We next stained the F-actin of the 3 groups of cells with rhodanmin-conjugated phalloidin to study the reorganization of F-actin bundles. Increased protrusion structures such as philopodia and lamelipodia were observed in *NTKL*-7703 cells, compared with parental QGY7703 cells (Figure 6B). In sh-*NTKL*-4 cells, F-actin staining was reduced and no protrusion structures were observed (Figure 6B). Since the small GTPases Rac1/cdc42 plays important roles in the formation of philopodia and lamelipodia (20), expression level of phosphorylated Rac1/cdc42 in sh-*NTKL*-1 and sh-*NTKL*-4 cells was checked by western blot. The results showed that level of phosphorylated Rac1/cdc42 was decreased in *NTKL* depleted cells compared with sh-control cells (Figure 6C). These findings suggested that overexpression of *NTKL* can facilitate philopodia and lamellipodia formation through regulating actin nucleation and phosphorylation of Rac1/cdc42.

### NTKL interacted with Dynamin2

In order to identify molecular mechanism of NTKL in regulating cell division and cell motility, co-immunoprecipitation assay was carried out using NTKL antibody as the bait, with rabbit IgG as negative control to identify interacting proteins of NTKL. Protein mixtures pulled down by NTKL antibody and IgG were then subjected to SDS electrophoresis. Specific bands showed in the experimental group but not in the control group were cut out and analyzed by mass spectrometry. Four potential NTKL-interacting proteins including Dynamin2, granulin, keratin and β-actin were pulled down by NTKL antibody (Figure 6D). Among these potential interacting proteins, Dynamin2 attracted our attention, for previous study showed that Dynamin2 might coordinate membrane remodeling and actin filament dynamics during cell morphogenesis and cell migration [18]. In addition, Dynamin2 has been localized to midbodies during cytokinesis [19]. The interaction between NTKL and Dynamin2 was further confirmed by co-immunoprecipitation assay (Figure 6E). Furthermore, we observed that NTKL and Dynamin2 co-localized with each other during cytokinesis by immunofluorescence staining (Figure 6F). These results indicated that the interaction between NTKL and Dynamin2 might be important for NTKL in regulating cell cytokinesis and cell motility. Further characterization of Dynamin2 might help elucidate the molecular mechanism of NTKL in tumorigenesis.

## DISCUSSION

Overexpression of *CHD1L* is one of the most frequent genetic alterations in HCC. The oncogenic function of *CHD1L* has been correlated with its upregulating downstream target genes such as *ARHGEF9* [8], *TCTP* [21], and *SPOCK1* [22]. In this study, we characterized another *CHD1L* regulated gene *NTKL* for its role in the development and progression of HCC. Our data showed that overexpression of CHD1L could upregulate the expression of *NTKL* in HCC cell lines. ChIP assay confirmed the binding of CHD1L to the promoter region of *NTKL*. In addition, expression of *NTKL* in HCC clinical samples was significantly correlated with that of *CHD1L* (*P* < 0.0001). All these results implied that *NTKL* might be a downstream target of CHD1L. Overexpression of NTKL was detected in 17.4% of HCCs, which was significantly associated with vascular invasion and poorer prognosis. Cox regression analysis showed that overexpression of *NTKL* was an independent factor for predicting poor 3-year survival of HCC patients (*P* = 0.033).

*In vitro* and *in vivo* functional studies found that NTKL had strong tumorigenic ability. Mechanism study found that overexpression of *NTKL* could accelerate G1/S transition, and the effect could be reversed by *NTKL* depletion. Although the exact mechanism of NTKL promoting cell cycle progeression was not clear, the accelerated degradation of P53 induced by NTKL would help explain the phenotype to some extent. In our previous study, we found that SCYL1BP1 (also called *NTKL-BP1*), a NTKL binding protein, could inhibit the Pirh2-mediated degradation of P53 [23], although the exact role of *NTKL* in this event has not been fully explored.

Another interesting finding of this study is that *NTKL* could accelerate the mitotic exit and chromosome segregation. The catalytic activity of Cdk1 is necessary and sufficient for maintaining the mitotic state of cells, and decreasing Cdk1 activity is the major factor to drive the exit of cells from mitosis [24]. One recent study finds that decreasing Cdk1 activity in mitosis causes a faster mitotic exit [25]. Using co-IP assay, we pulled down several potential NTKL-interacted proteins including dynamin2. Since the inhibition of dynamin2 can induce cytokinesis failure [26], dynamin2 might be involved in the regulation of mitosis [27]. Just like the results of inhibition of dynamin2, we found that depletion of *NTKL* could induce the cytokinesis failure, which subsequently led to the re-fusion of daughter cells and multipolar cell division, and finally triggered the apoptosis.

*Dynamin2* has been reported to participate to the regulation of actin filament assembly, filament stability and filament remodeling [28], which is related with many important process including cellular movements and cell division. Actin protein was translated as protein monomer called G actin, and G actin needs to be assembled into F-actin to function in cell movement and other process, and the process of this actin assembly was called actin nucleation [29]. *Dynamin2* takes part in actin nucleation by taking the hat off actin filaments to allow actin monomer to be assembled to the end of the actin filament thus resulted in the elongation of F-actin [29]. Two cellular structures related with F-actin assembly are filopodia and lamelipodia [30], which are frequently reported to form during epithelial-mesenchymal transition (EMT), and associated with tumor cell motility [31]. Dynamin2 has been also correlated with the degradation of extra cellular matrix at the front of protrusion [32]. In the present study, we found that overexpression of *NTKL* could increase cell motility by regulating F-actin reorganization. Actin nucleation was attenuated in *NTKL* depleted cells by the evidence of reduced F-actin staining. Downregulation of phosphorylated Rac1/cdc42 was also observed in *NTKL* depleted cells in accordance with previous report [20], that p-Rac1/cdc42 took part in the F-actin reorganization. Due to the fact that dynamin2 takes part in all the process mentioned above (e.g. cytokinesis, cell movements, actin nucleation and mitosis), and NTKL interact with dynamin2, we hypothesize that *NTKL* regulated cell motility, cytokinesis, actin assembly and mitosis through interacting with dynamin2. Further study is needed to fully understand the interaction among CHD1L, NTKL and dynamin2, as well as their roles and molecular mechanisms in the development and progression of HCC.

## MATERIALS AND METHODS

### Cell lines and tumor specimens

Human immortalized liver cell lines LO2, QSG-7701 and HCC cell lines, SMMC-7721, QGY-7703, CRL-8064 and MHCC-97H used in this study have been described in previous studies [7–9]. A total of 138 HCC patients, who underwent hepatectomy for HCC at Sun Yat-Sen University Cancer Center (Guangzhou, China), were included in this study. Human tissue samples used in this study were approved by the Committees for Ethical Review of Research involving Human Subjects at Cancer Center of Sun Yat-Sen University.

### Antibodies and western blotting

Western blot analyses were performed with the standard protocol. Antibodies for NTKL, dynamin2, p53, GAPDH, cyclinD1, phosphorylated threonine and β-actin were purchased from Abcam (Abcam, Cambridge, UK). Antibody for CHD1L was purchased from Genway (Genway Biotech, San Diego, CA). Antibodies for phosphor-Rac1/cdc42 were purchased from cell signaling (Cell Signaling Technology, Beverly, CA). Letin-88 and rhodanmine-phalloidine were purchased from Sigma-Aldrich (Sigma-Aldrich, St. Louis, MO).

### RNA extraction and quantitative real-time PCR

Total RNA was extracted using TRIzol Reagent (Invitrogen, Carlsbad, CA), and reverse transcription was performed using an Advantage RT-for-PCR Kit (Clontech Laboratories, Mountain View, CA, USA) according to the manufacturer's instructions. For qRT-PCR analysis, cDNA was amplified using a SYBR Green PCR Kit (Applied Biosystems, Foster City, CA) and an ABI PRISM 7900 Sequence Detector. Sequences of primers used in this study were listed in Supplementary Table 1. The relative fold change of NTKL was defined as fold increase of NTKL expression in the tumor tissues compared with their paired non-tumor tissues. The average fold increase of NTKL expression in HCC cases with NTKL up-regulation was 3.96, thus the threshold of 4-fold was chosen to define an overexpression in further analysis.

### Establishment of NTKL overexpressing and depleted cells

Full-length of *NTKL* was cloned into PCDNA3.1 and stably transfected into QGY-7703 (*NTKL*-7703) and MHC-97H cells (*NTKL*-97H) using lipofectamine2000 (Origene, Rockville, MD). Empty vector-transfected cells (Vec-7703 and Vec-97H) were used as controls. NTKL depleted cells were established by stably transfecting shRNA targeting NTKL (Origene, Rockville, MD) into QGY-7703 cells.

### Chromatin immunoprecipitation (ChIP)

The chromatin immunoprecipitation experiment was performed using an EZ-Magna ChIP^TM^ G kit (Upstate Biotechnology, Lake Placid, NY) according to previous studies [8].

### *In vitro* oncogenic assays

Cell growth rate was assessed by XTT kit (Roche Diagnostics, Indianapolis, IN), and colony formation in soft agar were carried out as described previously [6–8]. Both experiments were repeated independently in 3 times.

### Tumor xenograft mouse model

*NTKL*-97H and Vec-97H (4 × 10^6^) were subcutaneously injected to five-week-old nude mice (10 mice for *NTKL*-97H and 5 mice for Vec-97H), respectively. Tumor formation in nude mice was checked after 4 weeks. All animal experiments were done according to institutional standard guidelines in the Committee on the Use of Live Animals in Teaching and Research (CULATR) of the University of Hong Kong.

### Flow cytometry

Cells of 70~80% confluence were digested into single cell suspension, fixed in 70% ethanol, stained with propidium iodide (PI), and analyzed by flow cytometry. The results were analyzed with Modfit and Cell Quest software.

### Immunohistochemistry staining (IHC)

Tissue slides were dewaxed and dehydrated according to standard protocol. Antigen retrieval was done by boiling slides in antigen retrieval buffer for 15mins. Primary antibody was added onto the section and incubated at 4°C overnight. Secondary antibody was added for 1 hr after rinsing by PBS. DAB was used for staining.

### Co-immunoprecipetation and mass spectrometry analysis

CoIP assay was carried out according to manufacturer's instruction (Roche, Mannhein, Germany). Briefly, lysis buffer was added to NTKL-7703 cells and total protein was extracted by centrifuge. IgG or indicated antibodies were added into cell lysate and incubated for 1 hr on a rocking platform at 4°C, followed by addition of protein agarose G and incubation overnight. After washing, protein loading dye was added into protein agarose G containing the pulled down product and boiled at 100°C for 3 mins and subjected to SDS gel electrophoresis. After silver staining, bands appearing in the experimental group was cut off and sent for mass spectrometry analysis.

### Immunofluorescence (IF)

IF staining was performed as described previously (8). Briefly, cells seeded on cover slips were fixed by 4% paraformaldehyde and incubated with the indicated antibody overnight at 4°C. After washing, Texas-red or FITC conjugated secondary antibody was added to the cells and incubated for 1 hr. After washing, cells were counterstained with DAPI (Vector Laboratories, Burlingame, CA) and observed under a fluorescence microscope. For F-actin staining, after fixation as above, cells were stained with Rhodamine phalloidin (Invitrogen) for 30 mins.

### Realtime imaging

Cells were seeded onto 5-cm diameter CELL view dishes (Greiner Bio-One GmbH, Frickenhausen, Germany). Cell migration and cell cycle progression were observed under Perkin Elmer Spinning Confocal/Widefield Imaging system (Perkin Elmer, Waltham, MA). Time-lapse images were taken every 3 or 5 minutes for 48 hours with a 40 × objective lens. Cell migration was analyzed by Imaris software.

### Statistical analysis

The SPSS statistical package for Window version 13 (SPSS, Chicago, IL) was used for data analysis. RQ of NTKL in tumor and nontumor samples were compared using paired sample T-test. The survival curves of patients with or without NTKL upregulation was derived by Kaplan-Meier method. The correlation between clinical features of HCC patients and NTKL upregulation was carried out by Pearson χ2 test. A *P* value less than 0.05 was considered statistically significant.

## SUPPLEMENTARY TABLES


