# Supplementary material for: Overexpression of N-terminal kinase like gene promotes tumorigenicity of hepatocellular carcinoma by regulating cell cycle progression and cell motility
Source: Oncotarget. 2015 Jan 10;6(3):1618–30. doi: 10.18632/oncotarget.2730 (PMC4359319; doi:10.18632/oncotarget.2730)
Supplement: Supplementary file 1 [file oncotarget-06-1618-s001.pdf]

SUPPLEMENTARY TABLES

Supplementary Table 1: The DNA sequence of PCR primers

Name	Primer	Sequence	Note
NTKL	Forward	5'- CGGAATTCCGGGGGACGATGTGGTTCTTT -3'	Primers used for cloning NTKL
	Reverse	5'- CGGAATTCCGACGGTTCAGTCCAGCTTCC -3'	
RT- NTKL	Forward	5'- GCCTGTGCTCTGCGGTCT -3'	Primers used for QPCR
	Reverse	5'- GTGGCAGGAACAGGGGTG -3'	
RT-18s	Forward	5'- CTCTTAGCTGAGTGTCCCGC -3'	Primers used for QPCR
	Reverse	5'- CTGATCGTCTTCGAACCTCC -3'	
RT-CHD1L	Forward	5'- GGTGGAGTTGGCATGAACTT -3'	Primers used for QPCR
	Reverse	5'- CACTCAACTGGAGGTCAGCA -3'	
GFP-NTKL	Forward	5'- CGGGATCCCGGGGGACGATGTGGTTCTTT -3'	Primers for constructing GFP fused NTKL
	Reverse	5'- CGGAATTCCGTCCAGCTTCCGGGCTCCCA -3'	
NTKL-UP1	Forward	5'- CTTGCCTCCGCACCTA -3'	Primers used for Chip-PCR
	Reverse	5'- GCACAGGGTTGGAACTAAA -3'	
NTKL-UP2	Forward	5'- ATGGCTGGAGGTGAGATA -3'	Primers used for Chip-PCR
	Reverse	5'- CAGAACAGGGTGATGGTG -3'	

Supplementary Table 2: Cox proportional hazard regression analyses for 3-year survival

	Univariable analysis		Multivariable analysis	
**Clinicopathological features**	**HR(95%CI)**	***P***	**HR (95%CI)**	***P***
**NTKL expression**				
Without up-regulation	1		1	
With up-regulation	2.83(1.36–5.86)	***0.005***	2.47(1.08–5.68)	***0.033***
**Differentiation**				
Well differentiated	1		1	
Poorly differentiated	3.47(1.47–8.21)	***0.005***	3.16(0.98–10.21)	NS
**Vascular invasion**				
Absent	1			
Present	2.79(1.44–1.5.37)	***0.020***	1.29(0.29–5.68)	NS
**Tumor number**				
Single	1		1	
Multiple	2.29(1.29–4.06)	***0.005***	2.07(0.45–9.40)	NS

(*Continued*)

	Univariable analysis		Multivariable analysis	
**Clinicopathological features**	**HR(95%CI)**	***P***	**HR (95%CI)**	***P***
**Serum AFP level** [$\log_{10}$(ng/ml)]				
Low	1		1	
High	1.56(1.24–1.95)	***0.012***	1.94(1.25–3.03)	***0.003***
**Tumor stage (TNM)**				
Early	1		1	
Late	2.54(1.40–4.60)	***0.002***	1.32(0.28–6.21)	NS

CI = confidence interval; HR = hazard ratio; NS = not significant.
